# Faculty of Medicine of Paris—Lectures on General Pathology, by Professor Andral

**Published:** 1847-06

**Authors:** 


***Faculty of Medicine of Paris—Lectures on General Pathology, by Professor Andral.***—The seat of the morbid element by which disease is constituted may be sought for in three different parts of the system: 1, in the alterations of nervous influence; 2, in the changes of the blood; 3, in the changes which have supervened in those substances which emanate from the blood. In these three great groups many subdivisions should be introduced; and all diseases may either naturally, or with a little allowable hypothesis, be referred to one or the other order. Practically, however, we do not consider this classification applicable, and we prefer the following:—

We recognize fourteen great classes of diseases, viz.: 1, pyrexia; 2, hyperemia; 3, phlegmasia; 4, anemia, or hyperemia; 5, hemorrhage; 6, fluxes; 7, dropsy; 8, pneumatosis; 9, alterations of nutrition, or trophopathies; 10, gangrene; 11, traumatic diseases: 12, hemopathies; 13, alterations of secreted fluids, or crinopathies; 14, neuropathies.

A few words on each.

1. *Pyrexia.*—In this class the increase of temperature of the body is the only constant symptom; and pyrexiæ are also characterized by the factt hat it is impossible to trace them to any alteration of solid texture When such alteration appears during the progress of fever, they are caused by the same morbid influence which first occasioned the febrile excitement. They are specially observed on the skin and mucous membranes, and consist of congestion, inflammation, or hemorrhage—either interstitial in the shape of patechiæ and ecchymosis, or discharged from the affected surface; fluxes, as in cholera, miliaris, &c., are also observed. In each pyrexia the local alterations not only may have a special form, but seem to affect a special seat; the cellular tissue sometimes; the limphatic glands in syphilis; in other diseases the salivary glands; gangrenous inflammation of the cellular tissue and skin in the plague. The blood may be altered in its composition in pyrexia, and this alteration is always the same, consisting in a diminution of its natural amount of fibrine. In many pyrexiæ a virus or poison exists in the system.

Pyrexiæ are continuous or intermittent, hence a natural division into two orders: in the first we must form numerous genera, and amongst these we may name synocha, inflammatory fever, typhoid ever, miliaris, typhus castrensis, yellow fever, the plague, variola, rubeola, scarlatina, cholera, acute farcy, and carbuncular fever. These various maladies are connected with each other by their essential characters, and some arise spontaneously; others are developed by contagion or infection; and several are special to particular latitudes.

The second order is constituted by intermittent pyrexiæ, in some of which no other morbid phenomenon, beside the febrile excitement, can be detected: in others the febrile paroxysms are attended with various accidents, much the same as those which may be observed during the progress of continuous fever: fluxes, for instance, or losses of heat (which have not, however, been thermometrically proved,) but which have been known to cause death, as in the algid fever; hence a subdivision of intermittent fevers into benignant and pernicious. Cullen perfectly understood the connections between the two orders of pyrexiæ, so much so that as a type, in his description of fever, that illustrious author takes the paroxysm of ague. In the various parts of the globe, according to the intensity of the producing cause, of the different degrees of atmospheric heat, of the presence of marshes in mild or hot climates, we can observe the gradual transformation of intermittent into continuous fever, and reciprocally. They are different types of the same disease: but, like intermittent or continuous neuralgiæ, they are still the same malady.

Elevation of temperature is the only constant phenomenon which observation proves to exist in fever. Entering the field of hypothesis, may we not suppose this increase of heat to be the result of a more active combustion produced in the system by a temporary arrest of the nutritive movement? The removal from the system of a large quantity of nitrogen with the urine during fever, the production of an increased amount of water, carbonic acid, and bile in the same condition, for the separation of superabundant carbon and hydrogen, would seem to confirm our supposition. This arrest of the nutritive movement can occasionally be referred to the inference of a deleterious agent, external or internal, by which the vital properties are more or less interfered with, and the activity of nutrition slackened; or it may be attributed to an inflammation which calls to a limited part of the body an accumulation of blood, producing new secretions, a new mode of nutrition, a new and morbid elaboration of the elements of the blood; under the influence of a similar process, it is not impossible to admit that the general nutritive movement may be suspended, or at least rendered less active. All this is doubtless hypothetical; but a time comes when the mind, wearied with the dryness of the mere observation of facts, cannot resist the impulses of imagination; and such speculations, far from weakening its powers, serve, on the contrary, to enlarge its scope and to renovate its vigour.

2. *Hyperemia* is constituted by the accumulation of the blood in a part, due to the arrest or the diminution of the speed of its circulation; hyperemia is always attended with redness, sometimes with tumefaction; the serum of the blood, containing some albumen, but never any fibrine, may be extravasated during congestion. According to the nature of its causes, we divide this class into three orders: hyperemia due to increased excitability; hyperemia due to diminution of the powers of circulation; and hyperemia referrible to mechanical causes, impeding the return of venous blood to the heart.

3. *Phlegmasia.*—When congestion has been gradually transformed into phlegmasia, as the disease no longer remains limited to its solid seat; the blood is modified in its composition, and one of its constituent elements, fibrine, is increased in quantity. This latter circumstance is invariably connected with phlegmasiæ, when they are accompanied with febrile excitement; and yet fever alone will not produce it. We have never met with increase of fibrine in pyrexiæ. In most cases it is the first order of hyperemia which passes into inflammation, but the two others may also terminate in a similar manner.

4. *Anemia, or Hypohemia.*—This class refers only to local anemia, a disease observed only in a small number of organs—the brain and gastric mucous membrane, for instance.

5. *Hemorrhage.*—Of this class we will say nothing, having on a former occasion treated the subject *in extenso*. Hemorrhages may, like congestions, be divided according to their cause.

6. *Fluxes* are in most cases the result of appreciable organic alterations; in others, on the contrary, the discharge is the only pathological fact which can be detected. They are characterized by the similarity of the fluid secreted with that of the fluid eliminated during health by the same organ. Fluxes have, according to their seat, been divided into three orders:—1, fluxes of the skin. 2, of the mucous membranes; in this order some authors place cholera, and not without some good reasons; we prefer, however, classing that disease with pyrexiæ. 3' fluxes of glandular organs, primary or consecutive to congestion or phlegmasia.

7. *Dropsies.*—We mentioned on another occasion that all dropsies might be referred to one of two causes: an obstacle to the circulation of the blood, or a change of its composition. Dropsy in no case can, therefore, be considered as a primary fact—as anything but a symptom; but it is a symptom of such magnitude—it is a secondary fact of such immense practical importance—that we consider ourselves fully justified in forming for it a special class.

8. *Pneumatosis.*—In this class are placed all the gaseous accumulations within the cavities of our organs. We have reason to suppose that gases may even exist in the blood, and, whatever be their origin, they occasion symptoms and diseases which must have a place in all classifications.

We now come to the consideration of other diseases, in which the alterations of the solids is the primary pathological fact:—

9. *Trophopathies, or Diseases of Nutrition.*—These may be congenital or acquired; hypertrophy, atrophy, softening and accidental productions (subdivided into homologous, heterologous, parasitical,) and the various genera of acquired trophopathies.

10. *Gangrene* is in itself a disease which may be brought on by many causes besides congestion and inflammation. It may be produced by an obstacle to capillary or arterial circulation—by the introduction of miasmata or morbid poisons into the system, as in the plague. It is, therefore, something more than a termination of inflammation, and deserves to be separately studied in a class peculiar to itself.

11. *Traumatic affections* form, with the two preceding, the group of diseases in which alteration of the solids is primary.

12. *Hemopathies or Diseases of the Blood.*—The first order contains the diseases resulting from changes of proportion of the natural elements of the blood, and in this order we find four genera:—The first of which refers to the changes of proportion of the globules, which may be increased as in plethora, or diminished as in accidental or spontaneous anemia. In the second genus we place the changes of proportion of the fibrine—increased in inflammations attended with fever; diminished, on the contrary, and its diminution being a primary morbid fact, from which the other phenomena of disease may be derived—as in scurvy, or purpura;—or this dimunition being, as in pyrexiæ, not a cause but one of the possible results of the malady. In the third genus of the first order we place changes of proportion of the albumen in the blood; increase of the albumen has not hitherto been ever observed: its diminution is constant in one variety of dropsy. The fourth contains the changes of quantity noticed in the saline elements of the blood—a subject which has hitherto yielded to investigation only results of a negative nature.

The second order of hemopathies refers to diseases resulting from the introduction into the blood of principles not habitually contained in that fluid. This order is subdivided into three genera. The first, that in which the elements of our various secretions are found in the blood—for instance, tetanus; diabetes mellitus will probably, at a future day, be classed here. The second genus, that in which morbid products—like pus—are introduced into the blood. The third, in which it contains toxic principles, which may be modified in its constitution, or not changed in any appreciable manner. Here we must place diseases resulting from the passage into the blood of venous miasmata, virus of various sorts (rabies, syphilis, &c.,) or even mineral poisons, such as mercurial or saturnine emanations.

The third order of hemopathies is consecrated to asphyxia, in which, under the influence of various causes, the blood receives no oxygen, or an insufficient quantity of that gas.

13. *Alterations of secreted fluids* are mostly the result of other maladies; sometimes, however, they can be traced to no organic alteration whatever. Calculous affections of the bladder, kidney, and liver belong to this class, in which we must also place, for the present at least, diabetes mellitus. Albuminuria, when not connected with organic diseases of the kidneys, also should be classed here.

14. Neuropathies are primary disturbances of nervous action. They may be subdivided into the modifications of intellect, of sensibility, of motility, and of that nervous power which presides over the accomplishment of the functions of organic life.

Such, at the present day, is the most complete classification of diseases. This grouping into classes we have already said to be indispensable to the science of diagnosis, and to that of therapeutics.

*London Medical Times.*

